# Multidrug Resistant Epididymitis Progressing to Testicular Infarct and Orchiectomy

**DOI:** 10.1155/2013/645787

**Published:** 2013-11-27

**Authors:** Nicholas J. Farber, Rick C. Slater, Jodi K. Maranchie

**Affiliations:** ^1^University of Pittsburgh, Graduate School of Medicine, 401 Scaife Hall, 3550 Terrace Street, Pittsburgh, PA 15261, USA; ^2^Department of Urology, University of Pittsburgh Medical Center, 5200 Centre Avenue, Suite 209, Pittsburgh, PA 15232, USA

## Abstract

Global testicular infarction is a rare sequela of infectious epididymitis, with few reports in the urologic literature since the introduction of fluoroquinolones in the late 1980s. Ischemia occurs secondary to inflammation and edema of the spermatic cord with compression of arterial flow. We report a case of multidrug resistant epididymitis following prostate biopsy that progressed to global testicular infarction requiring orchiectomy. This case highlights the fact that epididymitis does not always follow an indolent pathway to resolution. Progression of pain should prompt early imaging and intervention. It further highlights the potential urologic consequences of the rising prevalence of multidrug resistant bowel flora in the United States, which will increasingly influence the management of presumed uncomplicated epididymitis, whether being primary or postprocedural.

## 1. Introduction

Bacterial epididymitis is one of the most common causes of scrotal pain in the United States [1]. Acute epididymitis typically presents with symptoms of epididymal swelling, induration, and exquisite tenderness to palpation and may also be accompanied by fever, chills, or dysuria [1]. History and physical examination alone are diagnostic but can be confirmed with positive urine culture and urinalysis findings of positive leukocyte esterase, presence of white blood cells, or by scrotal ultrasound.

The most common pathogens responsible for epididymo-orchitis vary with patient age [1]. Men younger than 35 are more commonly infected with sexually acquired organisms, including *C. trachomatis* or *N. gonorrhoeae*. Men over 35 typically demonstrate infection with enteric Gram-negative rods. Recent instrumentation with cystoscopy or transrectal ultrasound- (TRUS-) guided prostate biopsy also increases the likelihood of infection with Gram-negative rods [2]. Recently, multiple studies have shown an increasing prevalence of infection with fluoroquinolone-resistant strains of *E. coli* following TRUS-guided prostate biopsy, which can lead to potentially devastating complications due to ineffective treatment of epididymitis [3, 4].

Untreated epididymitis may progress to involve the testis, spermatic cord, or prostate. One of the most catastrophic complications is testicular infarction, which is thought to occur due to inflammation and edema resulting in compression of the testicular vein, artery, and lymphatics [5, 6].

## 2. Case Presentation

A 58-year-old male with a past medical history of diabetes mellitus presented with PSA of 7.8 and normal digital rectal examination. After counseling, he underwent in-office TRUS-guided prostate biopsy. He was given routine prophylactic periprocedural antibiotics with ciprofloxacin 500 mg by mouth in the morning of the procedure then Q12 hours for 2 additional doses. Three days after the biopsy, he developed pain in his right hemiscrotum. The pain progressed over the next several days without associated fever or chills. He presented to an outside emergency department on postprocedure day number 7 without contacting his treating urologist.

**Figure 1:**
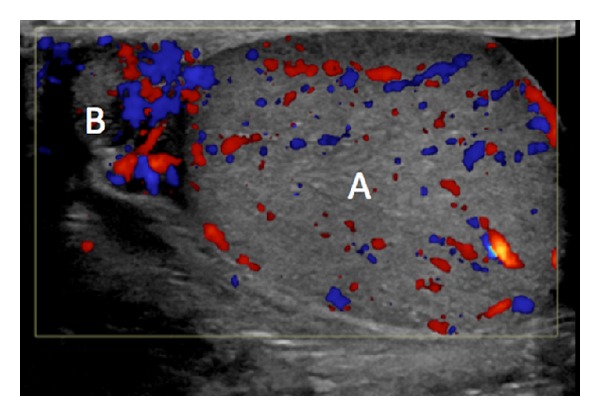
Right scrotal ultrasound with color Doppler interrogation at presentation to the ED showing A: right testis with preserved vascularity; B: right epididymis with enlargement and slightly increased vascularity.

On presentation, he denied other associated symptoms, including fever, chills, nausea, vomiting, hematuria, difficulty voiding, dysuria, change in bowel function, or urethral drainage. Physical examination revealed focal tenderness in the right epididymis, without erythema or fluctuance. He was afebrile and his white blood count was 11.4. Urinalysis revealed 2+ leukocyte esterase, positive nitrites, with 53 white cells per high power field. Scrotal ultrasound confirmed enlargement of the right epididymis with increased vascularity suggestive of acute epididymitis (Figure 1). He was given a single intramuscular dose of ceftriaxone in the emergency room, started on oral doxycycline, and admitted for observation.

Two days later, his white cell count had increased to 16.6. He remained afebrile but had persistent pain and swelling. Antibiotic coverage was changed to IV levofloxacin. On hospital day 4, the treating urologist was made aware of the admission. Exam at that time was grossly abnormal with a 6 cm firm, exquisitely tender right testis, substantial scrotal wall induration, and edema. Levofloxacin was discontinued and the patient was placed on broad coverage with intravenous ampicillin and gentamycin. Antibiotics were again changed to IV cefuroxime the following day when cultures grew *Escherichia coli* resistant to fluoroquinolones, ampicillin, sulfamethoxazole/trimethoprim, and doxycycline.

**Figure 2:**
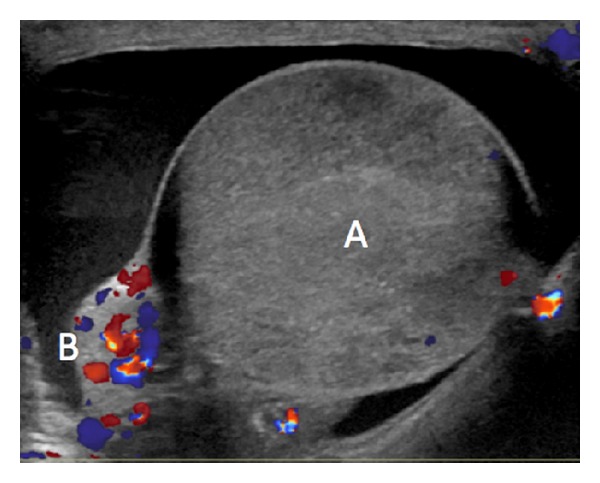
Right scrotal ultrasound with color Doppler interrogation on day 7 of hospitalization showing A: right markedly heterogeneous testis with blood flow essentially absent; B: prominent, hyperemic right epididymis.

**Figure 3:**
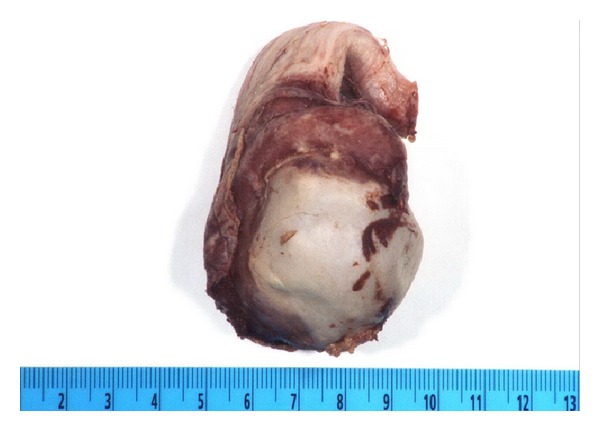
Orchiectomy specimen demonstrating indurated epididymis and spermatic cord with infarcted testis.

Despite this change his pain, swelling, and leukocytosis of 14.2 persisted and the patient continued to have low grade fevers on hospital day 6. Repeated scrotal ultrasound on hospital day 7 revealed complete absence of blood flow in the right testis consistent with global testicular infarct (Figure 2). Follow-up computed tomography of the pelvis and scrotum demonstrated no evidence of prostatic abscess. Due to persistent pain, fever, and radiographic evidence of testicular infarction the patient underwent scrotal exploration with right orchiectomy on hospital day 10 (Figure 3). He subsequently defervesced and his scrotal pain improved. He was discharged to home on four weeks of IV ceftriaxone. At most recent follow-up, his symptoms had resolved completely. Prostate pathology demonstrated Gleason 3 + 4 adenocarcinoma and he will pursue local therapy.

## 3. Discussion

We report a case of a 58-year-old gentleman undergoing a TRUS-guided prostate biopsy complicated by epididymitis, which was further complicated by antibiotic resistance of the offending organism resulting in progressive inflammation and edema, testicular infarction, and ultimately loss of the testicle. This case highlights management of postbiopsy infection as well as the rare entity of infectious testicular infarct.

Current guidelines for antibiotic prophylaxis in patients receiving TRUS-guided prostate biopsy suggest the use of a fluoroquinolone [7]. However, the incidence of gut colonization with fluoroquinolone resistant species is on the rise in many areas of the country, leading to increased incidence of postbiopsy infection and urosepsis. Qi et al. showed that 17% of patients undergoing prostate biopsy carried ciprofloxacin-resistant strains of *E. coli* and that 74.9% of the same ciprofloxacin-resistant strains were also resistant to ampicillin and ampicillin-sulbactam [4]. This trend has prompted some authors to suggest that stool cultures or rectal swabs are performed prior to prostate biopsy to guide antibiotic prophylaxis [3, 8]. Others have recommended alternative prophylaxis regimens including administration of fosfomycin [9], amikacin/ciprofloxacin combination [10], or augmentation with gentamycin [11]. Ultimately, choice of antibiotic coverage should be dictated by regional resistance patterns.

Although standard empiric therapy for suspected Gram-negative rods in epididymitis is a fluoroquinolone [1, 12], infectious prostatitis or epididymitis following a transrectal prostate biopsy that was empirically covered with fluoroquinolone must be assumed to be fluoroquinolone resistant. Initial therapy should consist of broad spectrum coverage until culture results are available to direct care. In the current case, the history of recent biopsy was not clearly conveyed to the admitting team resulting in a delay of starting appropriate antibiotic coverage. By the time effective antibiotics were given, vascular flow had already been compromised.

Epididymitis, being a very common cause of scrotal pain and inflammation, does not always follow an indolent pathway to resolution. Global testicular infarct secondary to infection is a rare sequela with few reports in the urologic literature since the introduction of fluoroquinolones in the late 1980s. Most recently, Yusuf et al. reported two cases of global testicular infarction with acute epididymitis [13]. Infarction is believed to occur due to spread of the inflammatory process to the spermatic cord resulting in compression of the testicular artery and decreased blood supply to the testis. Alternatively, development of a pyocele may cause extrinsic compression of the venous outflow and marked edema, which ultimately leads to loss of arterial flow to the testis [14]. Management of an infarct in the setting of infection requires scrotal exploration and orchiectomy in the majority of cases [15].

With increasing incidence of fluoroquinolone resistance it will be important to maintain a high index of suspicion for testicular infarct in the management of postprocedural and sporadic epididymitis. Progression of symptoms on therapy should prompt early imaging and surgical intervention.
